# Online Academic Cheating in Young Children and its Prevention

**DOI:** 10.1162/OPMI.a.381

**Published:** 2026-09-05

**Authors:** Minori Onishi, Yasuhiro Kanakogi

**Affiliations:** The University of Osaka, Osaka, Japan

**Keywords:** cheating, online, young children, visual perspective-taking

## Abstract

The global social situation changed dramatically after the COVID-19 pandemic, with various activities being shifted online. Some academic activities, including those for young children, are still conducted virtually. The transition introduced various challenges regarding academic honestly. However, whether young children engage in more academic cheating in online (vs. offline) settings remains unclear. Accordingly, this study examines this aspect, as well as how such behavior can be mitigated. We conducted two experimental studies with five-year-olds (*N* = 148), using a between-subjects design to examine children’s newly developed visual perspective-taking of proctors and their cheating behavior across offline, online, and two intervention conditions. In Study 1 (*n* = 74), we used the cost-benefit framework to investigate whether children would cheat more in online settings where proctors’ visual access is limited and the perceived costs of detection are consequently reduced. The visual perspective-taking task confirmed that most children recognized the limitations of online proctoring and were more likely to cheat online than offline. In Study 2 (*n* = 74), we used the Objective Self-Awareness theory to investigate whether real-time self-images would enhance self-awareness and reduce cheating compared to an avatar condition in which a human-like character tracked participants’ movements with a 2-second delay. Real-time self-images significantly reduced cheating in planned comparisons with both the online condition in Study 1 and an avatar control condition, though the omnibus test was marginally significant. Thus, young children are more likely to cheat online, where monitoring is limited, and fostering self-focused visual attention can reduce such dishonest behavior. These findings highlight the developmental foundations of online academic cheating and offer actionable strategies for improving young children’s online learning environments.

## INTRODUCTION

The coronavirus disease (COVID-19) pandemic substantially increased online communication, with many activities transitioning from face-to-face to virtual formats. The educational sector experienced a particularly significant transformation, with in-person instruction transitioning to online formats. In the context of early childhood education, the COVID-19 pandemic accelerated the global adoption of remote learning approaches. Many countries have actively promoted remote learning options for pre-primary education (UNESCO, 2020). In Japan, the Ministry of Education has encouraged preschools to implement remote and ICT-based education to sustain children’s learning and communication with teachers and families (Ministry of Education, Culture, Sports, Science and Technology (MEXT), 2021). National survey data from Japan indicate that over 80% of five- to six-year-olds already use internet-enabled devices daily (Children and Families Agency, 2025).

Although this transition enabled education to continue without major disruptions, it also introduced certain challenges, particularly regarding academic honesty. Research suggests that academic dishonesty increased following the onset of COVID-19 (Jenkins et al., 2023; Newton & Essex, 2024), and that cheating occurs more frequently in online university examinations than in traditional face-to-face exams (Janke et al., 2021). Notably, many digital learning tools designed for preschoolers include quiz-like tasks—such as multiple-choice questions or simple arithmetic problems—in which children must produce answers and receive feedback on correctness (e.g., Rüth et al., 2021; Taylor et al., 2022). As these assessment-like interactions become increasingly embedded in young children's daily learning experiences, understanding whether children are susceptible to cheating in such contexts has practical implications for the design of fair and trustworthy early childhood digital learning environments. However, little remains known about how online learning affects academic behavior in young children. In particular, although preschoolers have been shown to engage in cheating offline (Zhao et al., 2020, 2022), no studies have examined whether online educational settings increase the likelihood of dishonest behavior among this age group. One recent study examined children’s cheating behavior over Zoom (Dong et al., 2025). However, although the instructions were delivered online, the test itself was conducted without the experimenter present, as in prior offline studies, and no direct comparison was made between online and offline settings. Thus, whether children are more likely to cheat in online than in offline educational settings remains an open question.

Research on academic dishonesty in offline contexts has grown steadily, with prior studies showing that even young children engage in academic cheating (Zhao et al., 2020, 2022). For instance, Zhao et al. (2021) demonstrated that 5- to 6-year-old children cheated during a test, with more than half of the participants peeking at the answer sheet. They found that participants were more likely to cheat when the answer sheet was more clearly visible, suggesting environmental accessibility influences children’s dishonest behavior. Introducing a physical barrier between the child and the answer sheet reduced cheating, and even prompting children to imagine the presence of such a barrier produced a similar effect (Zhao et al., 2020). The results indicated that children were also less likely to cheat when the answer sheet was placed farther away, indicating that distance (between answer sheets and the children) influences academic cheating (Zhao et al., 2022). These suggest that specific situational factors can increase or decrease the likelihood of cheating among children, depending on the environmental context.

This, therefore, begs the question: Do young children engage more in academic cheating in online settings than in offline environments? The primary motivation for academic dishonesty appears to stem from a perception that the benefits outweigh the associated costs (Daty, 2022). For example, the potential benefits of cheating include obtaining good grades, experiencing less stress or anxiety, and saving time, whereas the costs include the perceived risk of detection and potential penalties (Miles et al., 2022). Critically, when the costs of an action decrease even as benefits remain constant, the cost-benefit balance shifts in favor of dishonest behavior. Online examinations may create precisely this scenario, reducing the costs associated with cheating. Recent research suggests that online examinations present relatively lower costs compared to face-to-face assessments (Newton & Essex, 2024). In particular, Newton and Essex (2024) revealed that the most commonly reported reason for cheating in university online exams was “opportunities to cheat”; namely, that cheating is easy and professors are unaware or do not care. Indeed, the detection of academic dishonesty in online contexts is difficult, potentially reducing the perceived cost for students and increasing the likelihood of dishonest behavior (Kajackaite & Gneezy, 2017; McCabe et al., 2001). Consistent with this reasoning, Becker et al. (2025) demonstrated that online-proctored examinations exhibit higher rates of cheating incidents compared to exams proctored in person. This can be understood through the cost-benefit theory (Drèze & Stern, 1987): when the perceived risk of being caught cheating is reduced, the expected penalties diminish, thereby lowering the costs of cheating. This lower perceived cost may shift the balance toward dishonest behavior, thereby increasing the incidence of cheating. Although online cheating among university students may be facilitated by various factors—such as access to unauthorized digital resources (Noorbehbahani et al., 2022), collaboration with peers via chat or messaging applications (Vaezi et al., 2024), and the limited visual access of online proctors (Atoum et al., 2017)—the last of these factors is most relevant to young children, who generally lack access to the technological means available to older students.

To address whether online settings with limited visual monitoring similarly lead to increased cheating in young children, the cognitive mechanisms that underlie dishonest decision-making in early childhood need to be examined. Prior developmental research suggests that at least two key processes must be operative (e.g., Sommerville et al., 2018; Zhao et al., 2025). First, for children to be sensitive to the cost of cheating, they must be capable of cost–benefit reasoning. Second, for children to perceive that cost as lower in online settings, they must recognize that the proctor’s visual access is constrained—that is, that the proctor cannot see the answer sheet. This recognition depends on visual perspective-taking ability.

The sensitivity to costs in behavioral decision-making, central to the cost-benefit framework, appears to have developmental roots in early childhood. Developmental psychology research indicates that even young children can consider costs and benefits when making behavioral decisions (Jara-Ettinger et al., 2016). Children as young as 10 months can infer costs from others’ actions and judge costly behaviors as more valuable (Liu et al., 2017). By 18 months, this sensitivity extends to children’s own behavior (Radovanovic et al., 2024; Sommerville et al., 2018). Studies examining objective costs—manipulated through block weight and number of steps required in carrying tasks—found that 18-month-old infants were less likely to help in high-cost conditions than in low-cost conditions. By age 5, children can integrate cost and benefit estimates, including social benefits such as reputation, to make behavioral decisions (Engelmann & Rapp, 2018). Indeed, 5-year-olds show sensitivity to observer-related costs in cheating contexts: they cheat least when an adult is directly observable, and most when neither visible nor audible (Zhao et al., 2025), and they are also influenced by reputational concerns (Zhao et al., 2018). Although these findings are consistent with cost-benefit reasoning, prior studies did not explicitly manipulate or define the relevant costs and benefits as such in theoretical terms. Moreover, it remains untested whether such sensitivity extends to online settings, where visual access is constrained by technological rather than physical factors. We hypothesize that this factor is closely linked to children’s visual perspective-taking abilities.

Theoretically, visual perspective-taking can be divided into two levels: Visual Perspective-Taking Level 1 (VPT1) and Level 2 (VPT2) (Flavell et al., 1981). VPT1 involves the ability to determine whether another person can see a particular object or scene, while VPT2 concerns the ability to understand that another person may view the same object or scene differently. In online settings, children must grasp that while they can see the answers, the proctor cannot. Thus, VPT2 is critical to understanding online cheating behavior in young children. Developmental research has shown that VPT2 typically emerges between the ages of four and five (Flavell et al., 1978, 1980; Frick et al., 2014). However, this body of work has focused almost exclusively on face-to-face contexts, leaving unanswered the question of whether children can apply VPT2 to camera-mediated monitoring.

If children do recognize that an online proctor’s visual access is constrained, they may estimate the cost of cheating to be lower than in offline settings—and, consequently, cheat more frequently. Whether these two capacities operate in online environments has direct implications for our understanding of children’s dishonest decision-making. Age 5 is thus a theoretically motivated starting point for this investigation: it is precisely the age at which VPT2 emerges and children show sensitivity to the costs of cheating, including both the risk of being observed (Zhao et al., 2025) and reputational concerns (Zhao et al., 2018). In Study 1, we investigated whether 5-year-olds understand online proctors’ visual limitations and whether cheating occurs more frequently online than offline. We hypothesized that children would cheat more in online settings—where the proctor's visual access is constrained—than in offline settings.

## STUDY 1

### Methods

Five-year-olds participated in a between-subjects experimental study in which they completed a newly developed visual-perspective-taking task and a challenging math test. In the offline condition (*n* = 37), a proctor monitored the children in person during the challenging task. In the online condition (*n* = 37), children were monitored online by a proctor. The research was approved by the Ethics Committee of The University of Osaka (approval number: R6-007). Informed consent was obtained from the legal guardians of all children. This study was not preregistered. All materials and anonymized data are available at https://osf.io/q8zta/.

#### Participants.

We predetermined the sample size per condition based on the average cheating rate for each experimental and control condition in prior studies that used similar methodologies (Zhao et al., 2020, 2021, 2022). Using estimated cheating rates of 53% and 22% for the online and offline conditions, respectively, a power analysis suggested that a sample size of 36 would suffice to detect a condition effect, with a power of .80 and an alpha of .05. Therefore, we recruited more than 36 children for each group. The 74 participants were equally split between the offline condition (*n* = 37; age: *M* = 64.78 months, *SD* = 4.06, range = 60–71 months; 18 girls) and the online condition (*n* = 37; age: *M* = 65.25 months, *SD* = 3.11, range = 60–71 months; 17 girls). Participants were recruited from a suburban city in Osaka, Japan, where most residents belong to the middle socioeconomic class. The sample comprised 95.9% ethnic Japanese, 2.7% of mixed ethnic heritage, and 1.4% of West Asian ethnicities. Seven additional children were tested but excluded from the analysis because they refused to participate (*n* = 2), discovered the hidden camera (*n* = 3), or made errors in a simple quiz during a practice test (*n* = 2).

#### Testing Room and Materials.

Participants were tested individually in a quiet laboratory. The room contained two tables and a chair: the participant was seated at the wider table (60 × 90 × 32 cm), with the narrower table (48 × 70 × 32 cm) positioned to their left. A digital countdown timer was placed on the wider table, and the answer sheet was placed on the narrower table, 0.6 m from the participant (Zhao et al., 2020, 2021, 2022). In the online condition, participants used the online communication software Zoom (Zoom Video Communications, Inc.) with a digital tablet (Microsoft Surface Pro 8) (Figure 1). In the pilot test (*n* = 7), we confirmed that all children could easily see the answer sheet while seated.

**Figure 1.**
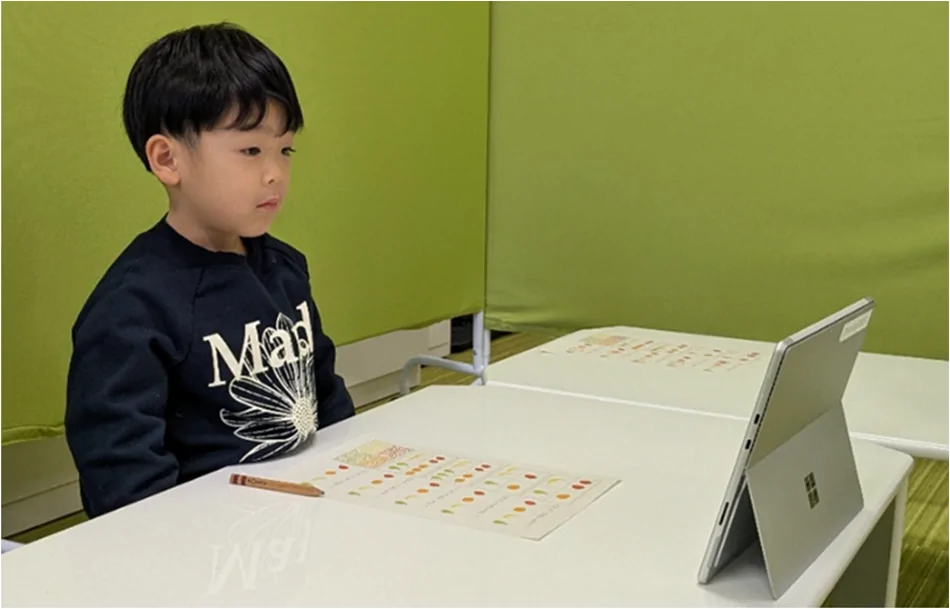
Environmental Setup of the Online Condition.

#### Procedure.

Each child was tested individually by two female experimenters: one provided instructions for all tests, and the other served as the proctor. In the offline condition, the proctor was present in the laboratory and interacted with the children in person during the tasks. In the online condition, the proctor interacted with the children online via Zoom while located in a separate room (Figure 2). The experiment proceeded in the following order: (1) introduction and warm-up game, (2) visual perspective-taking task, (3) practice test, and (4) main test. Before the main test, the instructor asked comprehension questions and told the children that she would place the answer sheet on the table. At that time, the offline proctor left the room and moved to the corridor, while the online proctor moved out of the camera's view. During the main test, the instructor also left the room and moved to the corridor. In the offline condition, the proctor was present in the laboratory and interacted with the children in person during the tasks. We recorded the children’s cheating behavior using a hidden camera and ensured they were unaware of its presence, as research shows that filming reduces cheating in 5-year-olds (Sai et al., 2024). At the beginning of the experiment, the instructor introduced herself and the proctor to the children. In the online condition, the instructor explained how online communication works: “This tablet device has a camera, and she (the proctor) can see you through it. She also uses a computer with a camera, and the image is shown like this [pointing to the screen].” The instructor showed the children a screen where the proctor was visible from the chest up. The children then played rock–paper–scissors three times with the proctor as a warm-up.

**Figure 2.**
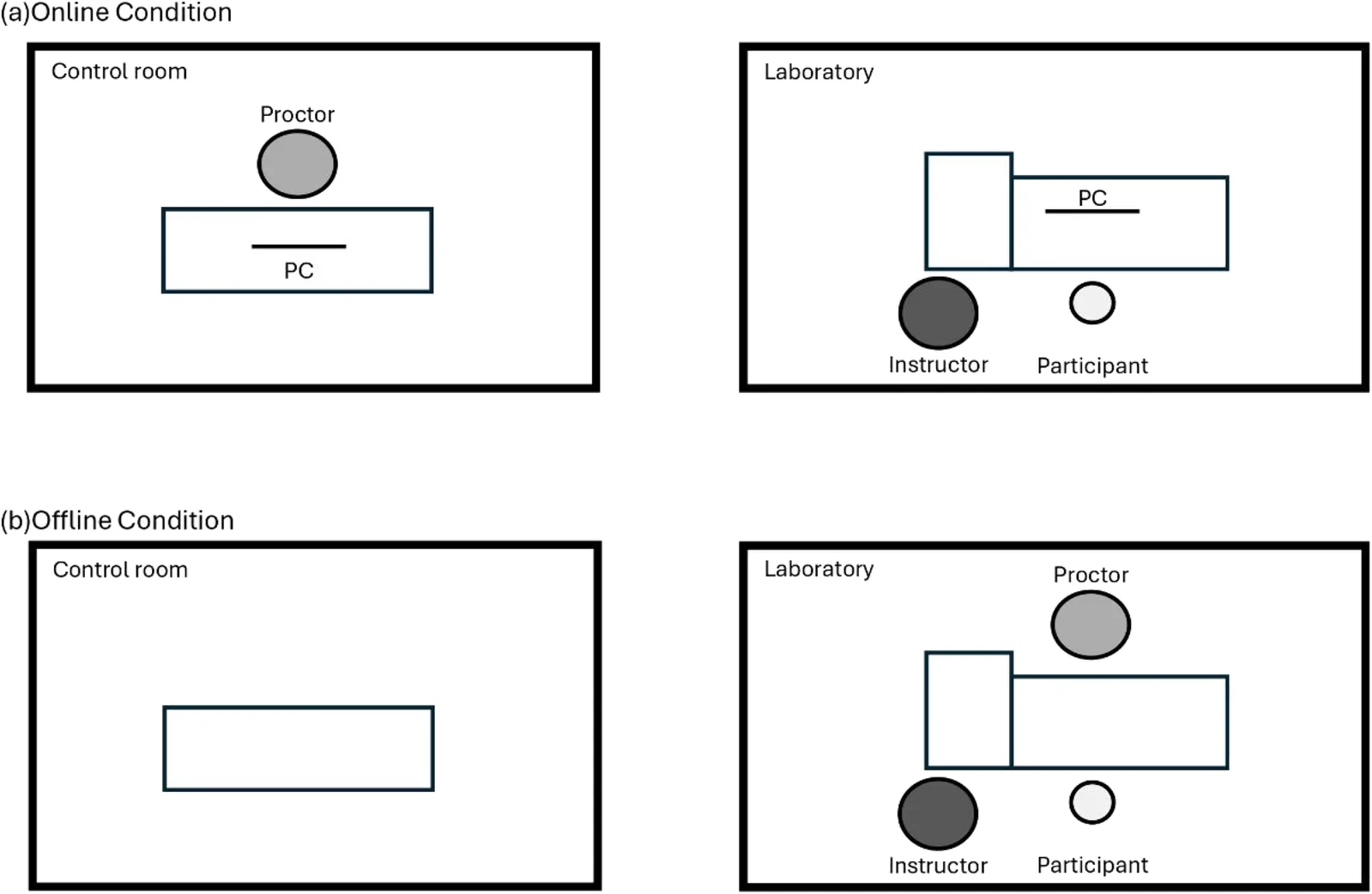
Experimental setup for online and offline conditions. *Note*. The online proctor interacted with participants from a separate room, while the offline proctor was present in the same room as the participants.

#### Visual-Perspective-Taking Task.

A prerequisite for applying the cost-benefit framework to children’s cheating behavior is that children recognize the differential monitoring constraints across contexts. To verify that children understood the key manipulation—that the online proctor had limited visual access compared to the offline proctor—we examined whether children understood that the online proctor could not see the location where the answer sheet was, whereas the offline proctor could. Additionally, because no previous research has investigated perspective-taking in online communication or compared it with offline interactions, we also explored children’s understanding of the proctor’s visual field for other areas around the child.

We divided the area around the seated child into six parts: face, chest, right side of the table, center of the table, left side of the table, and under the table (see Figure 3). The left side of the table was where the answer sheet was placed. The online proctor could see only the children’s faces and chests, while the offline proctor could see all areas except under the table. The instructor held a stuffed toy in each area in turn and asked the children, “Can she (the proctor) see this?” Children were not told whether their answers were correct or incorrect. These questions were posed in four different orders (2 × 2: at face-level or under the table, from the right side or the left side), counterbalanced across participants.

**Figure 3.**
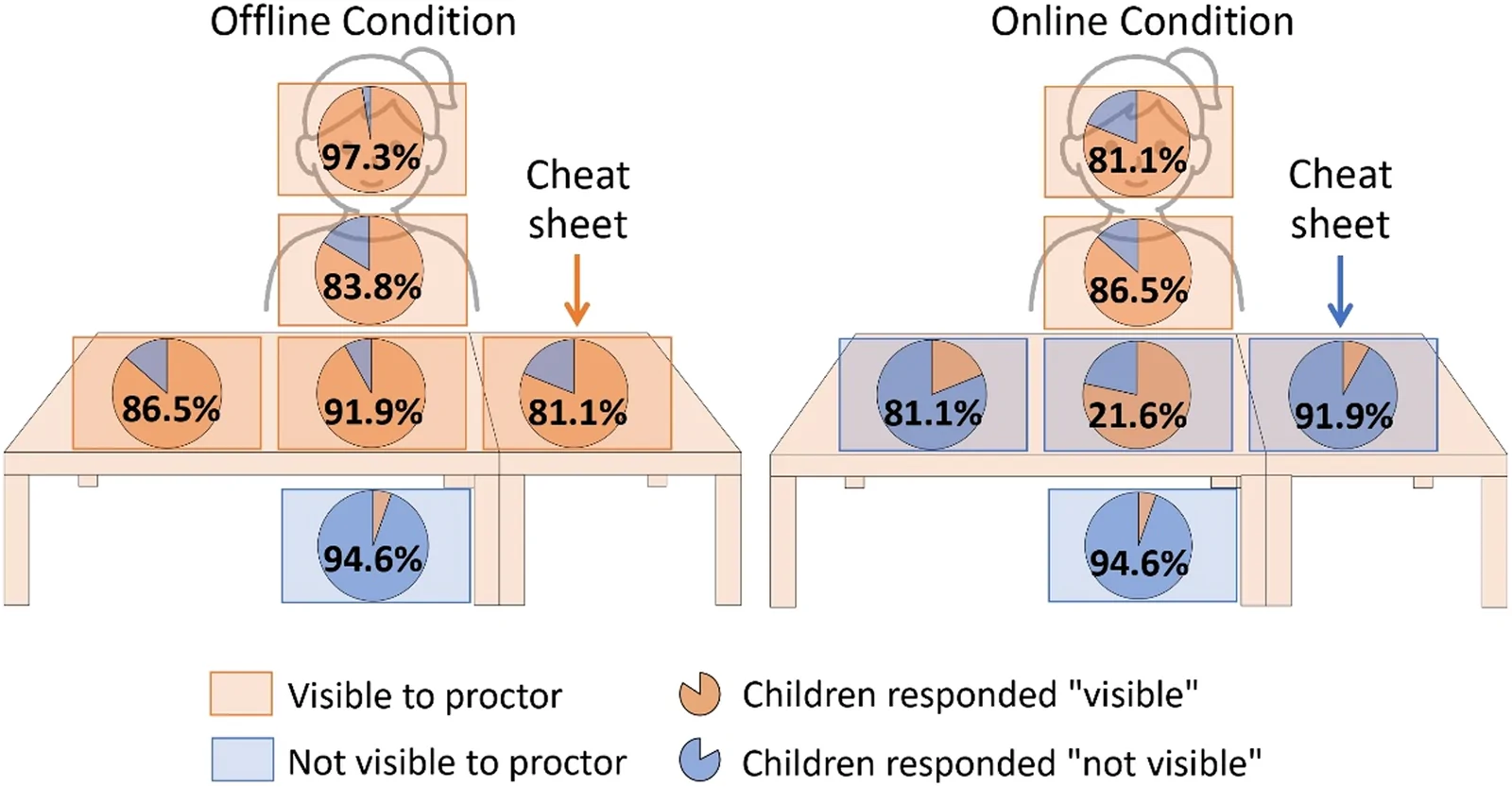
Correct answer rates for which areas could be seen by the proctor in the offline and online conditions. *Note*. The background color of the square indicates the actual visibility of each area to the proctor (orange = visible; blue = not visible). Within each pie chart, orange represents the proportion of children who responded “visible” and blue represents the proportion who responded “not visible.“ The numerical value displayed indicates the percentage of children who correctly judged whether each area was visible to the proctor.

#### Test Phase.

We conducted a warm-up, a practice test, and the main test, based on previous studies (Yee et al., 2024; Zhao et al., 2022). In the warm-up, children answered three easy questions about animals. After the instructor gave the children a question paper, the proctor read each question aloud, and the children were instructed to circle the correct answers. After checking the children’s answers, the instructor emphasized that they had chosen the right answers and drew a circle (which signifies a correct answer in Japan) on the paper. In the practice test, children answered three questions involving counting fruits in four squares and determining which square contained the most number of fruits. These questions were easy for 5-year-olds, and all but two participants selected the correct answers. The two children were excluded from the analysis because the practice assumed children could respond correctly without referencing the answer sheet (Zhao et al., 2022). As in the warm-up, the instructor checked the children’s answers and drew a circle on the paper to acknowledge their correct responses.

The main test was conducted in the same manner as the practice test, but combined four easy questions with one difficult question that no child in the pilot test was able to answer correctly within the time limit (Yee et al., 2024). To verify that items functioned as intended, we computed accuracy rates across all participants. The four easy items were answered correctly by 97.3%, 95.9%, 94.6%, and 98.6% of children, respectively. The difficult item was answered correctly by 44.6% of children (including those who cheated), confirming that it was substantially more challenging than the easy items. The instructor explained, “You will have up to five minutes to finish the test. Here is a clock [putting the countdown clock on the table] that will show you how much time is left for you to work on the test. Your test cannot be scored if you don’t finish on time.” Subsequently, the instructor made an excuse to leave the room, saying, “Oh, I just realized I forgot to do something I had to do!” The proctor told the instructor, “The participant can work on the test with me, so you can go to finish the job. I will call you when the test is done.” The instructor agreed with the suggestion, and the proctor left the room (screen) in the offline (online) condition to retrieve her cell phone so that she could call the instructor when the test was finished. The instructor repeated the test rules and asked participants to tell the proctor when they had finished the test. She also said, “I will put an answer sheet on this table. I will score your test by using this. Don’t peek at the answer sheet.”

To check comprehension, the instructor then asked each participant: (a) How long do you have to finish the test? (b) What should you do when you finish the test? (c) What will happen if you do not finish the test within five minutes? If a child answered incorrectly or did not respond, the instructor repeated all the instructions and asked the questions again. All children answered correctly the first or second time. When the comprehension checks were completed, the proctor returned to the room (screen) in the offline (online) condition with her cell phone. The instructor started the timer, placed the answer sheet on the table to the left of the child, and exited the room. Notably, the offline proctor could see the answer sheet, whereas the online proctor could not. This setup was designed to create a situation where detecting dishonest behavior would be more difficult in the online condition than in the offline condition (Becker et al., 2025; Newton & Essex, 2024).

Each child completed the test within 5 minutes as instructed and informed the proctor when they had finished. When summoned by the proctor, the instructor re-entered the room and checked the answers. After completing the test, all children were praised for their work, regardless of whether their answers were correct or incorrect.

#### Coding.

Cheating behavior was defined as children peeking at the answer sheet and copying answers from it in the instructor’s absence, but in the proctor’s presence, offline or online. We coded cheating behavior based on video recordings captured by a hidden camera. We reviewed all the children’s videos and answers to identify which children had answered correctly by looking at the answer sheet. Children who looked at the answer sheet but did not copy the answer (i.e., retained their original incorrect response) were not coded as cheating. Children who provided the correct answer but did not look at the answer sheet were also not coded as cheating. Coding was performed by two coders, and 100% of the cheating data were double-coded. Intercoder reliability was 100%.

### Results

A single binomial generalized linear model was fitted with condition, sex, and age (in months) as predictors to assess potential covariate effects. The analysis revealed no significant main effects or interactions involving gender or age (all *p*s > .10). Accordingly, the GLM reported below included only condition as the predictor.

#### Comprehension of the Proctor’s Vision.

The visual-perspective-taking tests confirmed that the children understood the proctors’ limited vision. In the offline condition, 81.1% of children correctly answered that the offline proctor could see the left side of the table (where the answer sheet was placed). In the online condition, 91.9% of children correctly answered that the online proctor could not see the left side of the table. These results indicate that most children understood that the proctor could see the answer sheet offline but not online.

Regarding other areas, most children in the offline condition responded correctly across all the other five areas (97.3%, 83.8%, 86.5%, 91.9%, and 94.6% for face, chest, right side, center, and under the table, respectively; see Figure 3). Similarly, children in the online condition answered accurately for all areas except one (81.1%, 86.5%, 81.1%, 21.6%, and 94.6% for face, chest, right side, center, and under the table, respectively). Notably, most children incorrectly believed that the online proctor could see the center of the table, even though she could not.

#### Cheating.

Figure 4 shows that the cheating rate in the online condition (35.1%) was higher than that in the offline condition (8.1%). To examine the effects of the experimental condition on cheating occurrence, we fitted a generalized linear model (GLM) analysis with a binomial distribution and a logit link function. Cheating occurrence (0 = no cheating, 1 = cheating) served as the dependent variable, and condition (offline condition = 0, online condition = 1) was the independent variable. The analysis revealed a significant effect of condition (*χ^2^*(1) = 8.47, *p* = .004, *OR* = 6.14, 95% CI [1.75, 28.94], two-tailed; *α* = .05). These results indicate that the children in the online condition were substantially more likely to cheat than those in the offline condition.

**Figure 4.**
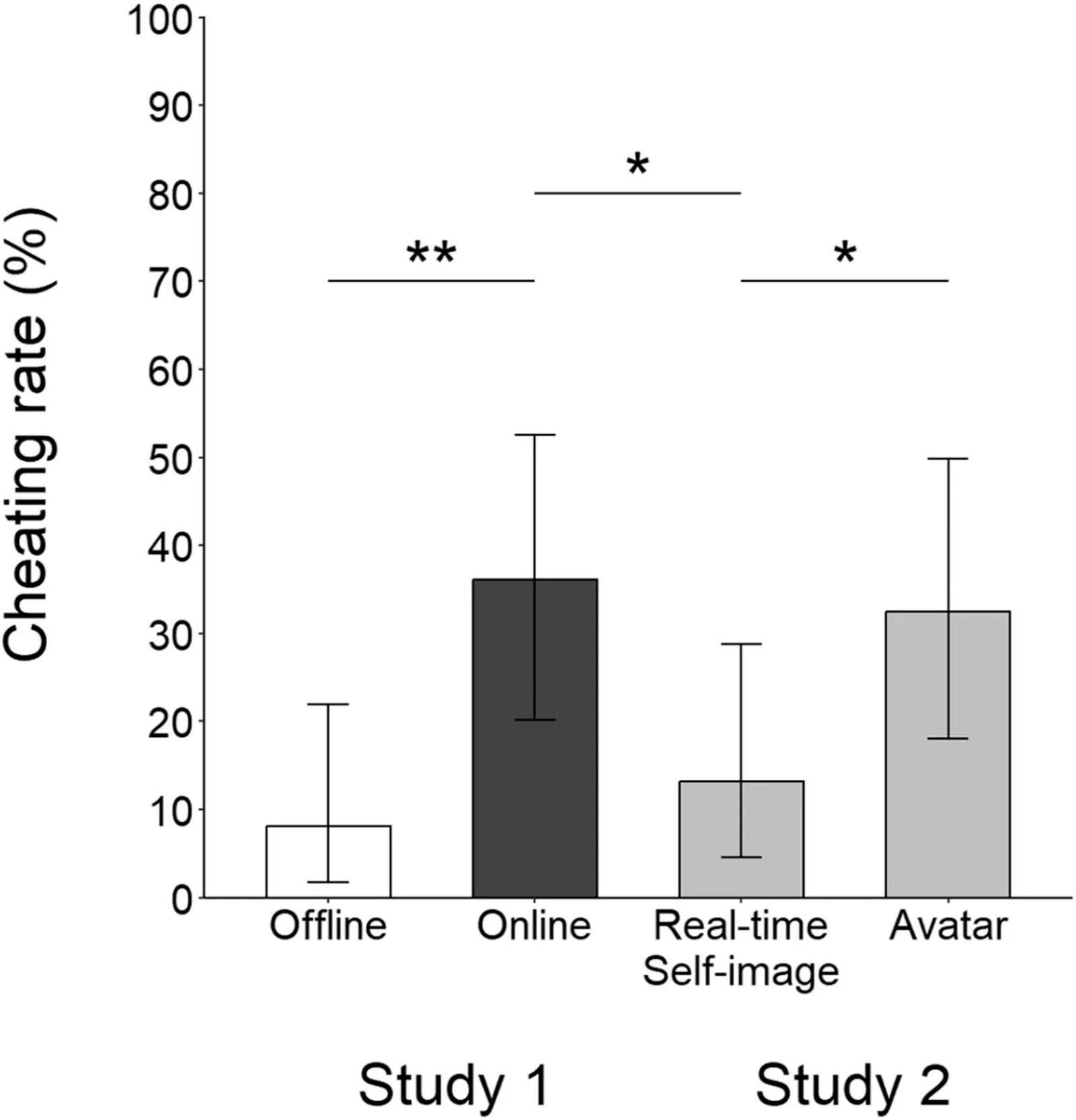
Cheating rates across Study 1 and Study 2. *Note*. **p < .01, *p < .05; error bars: 95% CI.

Given that the online condition was designed to create a situation with limited visual monitoring, and that children were expected to understand this limitation, we conducted a more stringent analysis. Specifically, we analyzed only those children who correctly identified the left side of the table in the visual-perspective-taking test, thereby confirming their ability to understand the proctor’s limited visual perspective. This resulted in a subset of 64 children (34 in the online condition and 30 in the offline condition). This approach allowed us to examine the effect of the condition using only participants who could potentially perceive the reduced cost of cheating when the answer was not visible to the proctor.

A binomial GLM with the same structure as the previous analysis revealed that the effect of condition remained significant among this subset of participants (*χ^2^*(1) = 7.24, *p* = .007, *OR* = 5.57, 95% CI [1.56, 26.65], two-tailed; *α* = .05). The cheating rate in the online condition (38.2%) was significantly higher than that in the offline condition (10.0%). These results confirm that children’s cheating increases in online situations when there are constraints on monitoring.

### Discussion

Study 1 tested whether children understood the proctor’s visual perspective and whether the online condition promoted children’s cheating more than the offline condition. The results demonstrated that children correctly judged the online proctor’s view as more limited than the offline proctor’s view, and they were more likely to cheat in the online than the offline condition. This indicates that children comprehend the limited monitoring that is possible online, which encourages them to cheat in the online condition.

The present study focused on children’s understanding of visual perspectives in online interactions. While extensive research has examined visual perspective-taking in face-to-face contexts (Flavell et al., 1978, 1980; Frick et al., 2014), little is known about whether children can understand the perspective of others in online settings, where visual information is mediated through screens and cameras. Our findings demonstrate that 5-year-old children can recognize an online proctor’s restricted view, representing the first evidence of children’s understanding of camera-mediated visual perspectives in educational contexts. Moreover, the perspective-taking task revealed that most children correctly identified the majority of areas visible or invisible to proctors in both offline and online conditions. This represents an important contribution to our understanding of children’s social-cognitive development in an era where digital communication has become increasingly central to educational and social experiences. Furthermore, while previous research on children’s cheating behavior has focused exclusively on offline educational settings (Zhao et al., 2020, 2021, 2022), the present study is among the first to examine academic cheating in online environments among young children. Beyond documenting this applied effect, the findings advance theory in two respects. First, they extend VPT2 theory by demonstrating that this capacity is not limited to face-to-face contexts: children were able to infer another person's visual access even when it was mediated by camera technology—a case where the boundaries of visibility are not physically apparent but must be imagined based on an understanding of how cameras work. At the same time, the finding that children systematically misjudged the online proctor's view of the center of the table suggests that camera-mediated perspective-taking presents unique challenges beyond traditional face-to-face contexts, pointing to an important boundary condition of VPT2. One possible explanation is that the linear area connecting the proctor and the child was closest to the screen and appeared in front of the proctor (Figure 1), leading children to believe that the center of the table was visible. These findings suggest that children may require specific experience or instruction to fully grasp the limitations of camera-mediated views. Second, the higher rates of cheating observed in the online condition, where the proctor's visual access was constrained, are consistent with the predictions of the cost-benefit framework, suggesting that young children's dishonest decision-making may be sensitive to monitoring constraints even in technologically mediated contexts.

Our findings indicate that online settings may create conditions that facilitate cheating behavior, making it crucial to develop interventions that prevent such behavior in early childhood contexts. Given that the external deterrents of cheating—particularly the risk of detection through visual monitoring—are generally lower in online than offline settings, we reasoned that increasing the internal deterrents to cheating may offer a promising intervention approach. One theoretical framework that addresses such internal deterrents is Objective Self-Awareness (OSA) theory. Consistent with this theory, children who viewed themselves in a mirror showed reduced cheating in a gift-delay task (Martin et al., 2020) and told fewer lies (Bender et al., 2018). OSA theory posits that when conscious attention is focused inward, people tend to engage in self-evaluation and attempt to reduce perceived discrepancies between their behavioral standards and their actual behavior (Duval & Wicklund, 1972). This inward focus can be induced by the introduction of various stimuli, including mirrors, cameras, or an audience (Duval & Wicklund, 1972; Silvia & Duval, 2001). Specifically, dishonest behavior may be prevented because directing attention to oneself through mirrors or cameras enables individuals to recognize the discrepancy between a standard that they should not engage in deceptive practices and their actual intention to do so. This recognition creates motivation to modify their behavior to align with their ethical standards, thereby reducing the discrepancy and preventing dishonest acts.

Similar intervention strategies have been applied in computer-mediated communication contexts. For instance, real-time video self-images increase adults’ self-focus (Sohn et al., 2019). For individuals to perceive discrepancies between their behavioral standards and actual behavior, two prerequisites must be met: they must have internalized the relevant standard and be able to recognize themselves in mirrors or videos. Research has indicated that even preschool children have internalized the standard that one should not engage in cheating behavior (Schmidt et al., 2016; Schmidt & Tomasello, 2012). Furthermore, children begin to develop self-recognition by 18 months of age, and by age 5, they can reliably identify themselves when viewing video recordings of their own behavior (Rochat, 2003). Therefore, presenting a real-time self-image may serve as an effective intervention to reduce cheating behavior in preschoolers.

## STUDY 2

In Study 2, we examined intervention strategies for preventing online cheating by using real-time self-images to increase participants’ self-awareness. We modified the online condition from Study 1 and conducted experiments with two newly established experimental groups. Both conditions were identical to the online condition in Study 1, where participants were monitored virtually, except for the addition of video elements displayed on the screen.

The first condition was a group exposed to a real-time self-image designed to promote self-awareness. A real-time video of the participants was displayed in the upper right corner of the online communication screen. This approach was based on the premise that focusing attention on oneself during tasks can prevent cheating behavior (Martin et al., 2020). The second condition was a group exposed to an avatar that served as a control to determine whether differences between the real-time self-video condition and Study 1's online condition resulted specifically from self-imagery. Instead of displaying the participant’s real-time self-video, this condition presented an avatar on the online communication screen.

For self-awareness to be promoted through self-image, participants must recognize themselves in the videos. Therefore, the avatar was designed to reduce self-recognition. Self-recognition is known to increase when individuals experience higher levels of agency and ownership toward the person depicted in the video (Kim et al., 2020). Dewe et al. (2025) demonstrated that agency and ownership decrease when an avatar’s appearance and movements are asynchronous with the participant, compared to when they are synchronized. Therefore, we presented an avatar whose appearance and movements were deliberately unsynchronized with the participants to prevent self-recognition.

Based on OSA theory, we hypothesized that compared to the online group in Study 1, the group exposed to the real-time self-image condition would exhibit lower rates of cheating behavior due to enhanced self-awareness. In contrast, the group in the avatar condition would demonstrate higher rates of cheating behavior than the real-time self-image condition because self-awareness would not be promoted.

### Methods

#### Participants.

Similar to Study 1, the new sample comprised 74 five-year-olds, divided equally between the real-time self-image condition (*n* = 37, age: *M* = 64.74 months, *SD* = 3.59, range = 60–71 months; 17 girls) and the avatar condition (*n* = 37, age: *M* = 65.62 months, *SD* = 3.60, range = 60–71 months; 19 girls). Participants were recruited from a suburban city in Osaka, Japan, where most residents belong to the middle socioeconomic class. The sample comprised 98.6% ethnic Japanese and 1.4% of mixed ethnic heritage. One additional child in the real-time self-image condition was tested but excluded from the analysis because they made errors in the simple practice quiz. This study was not preregistered. All materials and anonymized data are available at https://osf.io/q8zta/.

#### Materials.

The experimental setup was identical to that in the online condition in Study 1, except that the screen showed children either a real-time video image of themselves or a generic child avatar. The real-time video was captured using a laptop camera and was displayed in the upper right corner of the screen at one-sixteenth the screen size. The avatar was a human child character generated using Zoom's built-in avatar feature and displayed via OBS Studio, which introduced a two-second delay between the participant's movements and the avatar's corresponding movements. Each avatar was selected to correspond to the participant's gender. We matched appearance and synchronized movement between the real-time self-image and the avatar to isolate the effect of self-focus.

#### Procedure.

The procedure was identical to that of Study 1, except for the added explanation of the image or avatar before the warm-up game. After telling the children that the tablet had a camera and the proctor could see them, the instructor drew the children’s attention to the video by asking them to confirm that either themselves or their avatar was displayed on the screen. The instructor said, “Look! This is how she sees you [pointing to the real-time image]” in the real-time self-image condition, and “Look! This is a character [pointing to the avatar],” in the avatar condition. To prevent participants from self-identifying with the avatar, it was presented as a character without disclosing that the avatar’s movements corresponded to their own. Additionally, to verify whether participants self-identified with the avatar, we administered a post-experiment question using age-appropriate language: “Did you notice anything about the character on the screen?” Importantly, no child perceived the on-screen character as their own body (ownership) or as something they could control as intended (agency), and no child recognized the on-screen character as a representation of themselves.

#### Coding.

The dependent measures were identical to those in Study 1. We reviewed all videos and answers to identify which children had answered correctly by looking at the answer sheet. Coding was performed by two coders, and 100% of the cheating data were double-coded. Intercoder reliability was 100%. For the three remote conditions (the online condition from Study 1, the real-time self-image condition, and the avatar condition), we measured gaze duration—defined as the cumulative period of time during which children were coded as looking at the screen (as opposed to looking away)—from the start of the timer to the moment the instructor returned to the room. The primary coder coded all gaze duration data, and the secondary coder coded 25% of the data (*n* = 28). Intercoder reliability was 94.6%.

### Results

In Study 2, we investigated whether increasing self-awareness through visual feedback could reduce cheating behavior in online settings. We conducted the experiment with two new online groups: a real-time self-image group, in which participants viewed their own live video to enhance self-awareness, and an avatar group, in which an unsynchronized avatar with a 2-second delay was shown instead of the participant’s self-image to control for the effect that depended specifically on self-recognition.

Figure 4 shows that the cheating rate in the real-time self-image condition (13.5%) was lower than that in the online condition in Study 1 (35.1%) and the avatar condition (32.4%). To examine the effects of experimental conditions, we fitted a GLM with a binomial distribution with a logit link function. Cheating occurrence (0 = no cheating, 1 = cheating) was the dependent variable, and condition served as the independent variable (online condition = 0, real-time self-image condition = 1, avatar condition = 2). The overall model was marginally significant (*χ^2^*(2) = 5.64, *p* = .060, two-tailed; *α* = .05) on cheating occurrence. Given our hypothesis that self-focused attention reduces cheating, we conducted two planned one-tailed comparisons using the Holm correction for multiple comparisons. Specifically, we tested whether the real-time self-image condition showed lower cheating rates than (1) the online condition from Study 1 and (2) the avatar condition.

Participants in the real-time self-image condition were significantly less likely to engage in cheating than those in the online condition (*OR* = 3.47, 95% CI [1.18, 10.2], *p* = .036, one-tailed, Figure 4). Participants in the avatar condition also cheated more than those in the real-time self-image condition (*OR* = 3.07, 95% CI [1.05, 8.98], *p* = .036, one-tailed). These results suggest that exposure to the real-time self-image effectively reduces cheating behavior. More broadly, they indicate that the intervention’s effectiveness became apparent specifically when the children viewed themselves in real-time, underscoring the importance of self-focused attention in reducing academic cheating.

Importantly, gaze duration did not differ significantly across the three remote conditions (38.88 s for the online condition, 30.65 s for the real-time self-image condition, and 38.10 s for the avatar condition). Because the gaze duration data were not normally distributed, we fitted a Gamma GLM with a log link. This analysis revealed no significant main effect of condition, *χ^2^*(2) = 5.61, *p* = .061, two-tailed, indicating that the observed differences in cheating behavior were not attributable to differences in attention to the screen.

### Discussion

We conducted Study 2 to examine intervention strategies for preventing online cheating. A three-group comparison revealed a marginally significant effect of conditions. The marginal omnibus effect may partly reflect limited statistical power, as the sample size, which was originally calculated for a two-group design, was determined to match that of Study 1 for direct comparison. However, planned comparisons between the real-time self-image and the online conditions, and between the real-time self-image and the avatar conditions revealed significant differences in both cases. The results showed that children were less likely to cheat in the real-time self-image condition than in the online condition from Study 1. Additionally, children in the avatar condition were more likely to cheat than those in the real-time self-image condition. The only difference between the two groups was whether the images displayed on the screen were their own or an avatar. Therefore, these findings suggest that real-time self-image reduced online cheating behaviors, and this effect was likely caused by focusing children’s attention on themselves.

Our results are consistent with previous research, which indicates that self-awareness can reduce cheating behavior in young children (Bender et al., 2018; Martin et al., 2020). Notably, prior work has examined self-awareness as an honesty-promotion technique primarily in Western samples (Bender et al., 2018; Martin et al., 2020) and in Chinese children (Ding et al., 2019). The present findings extend this evidence to a Japanese sample, suggesting that self-focused attention may reduce dishonest behavior across a broader range of cultural contexts.

The present study also differed from prior research in the method used to induce self-awareness. We used a real-time self-image rather than a mirror. While previous studies with adults have demonstrated that presenting real-time video enhances self-awareness (Sohn et al., 2019), this has not been investigated in children. Our experiment is the first to examine whether exposure to a real-time self-image video is effective in increasing children’s self-awareness and suppressing cheating behavior. This change in the type of self-image was accompanied by corresponding changes in the size of the self-image and in the techniques used to sustain self-focused attention. Previous experiments using mirrors have typically employed large mirrors or incorporated additional methods to enhance self-awareness while children viewed their reflections (Bender et al., 2018; Ding et al., 2019; Martin et al., 2020). For example, children were asked to say their own names or point to parts of their own bodies in the mirror (Bender et al., 2018; Ding et al., 2019). In contrast, our display was smaller and required no additional techniques to draw attention. The effectiveness of our method may be attributed to characteristics unique to real-time self-images. In our experiment, real-time self-images were displayed in the upper right corner of the computer screen. This position potentially made the self-image more accessible than a mirror.

Another difference between our study and previous research lies in the rigor of the control condition. In previous studies, self-awareness was manipulated by placing a mirror in the experimental setting, with control conditions involving either covering the mirror (Martin et al., 2020) or turning it around (Bender et al., 2018). However, this approach introduced confounding differences in the presence or absence of visual stimuli. In contrast, our study presented visual displays on the screens in both conditions. This method allowed us to control for the amount of visual stimulation and movement characteristics while manipulating only the focus on the “self.” Therefore, we were able to provide more rigorous evidence than previous research suggesting that self-awareness plays an important role in reducing cheating behavior. It should be noted, however, that the avatar condition may not constitute a perfectly neutral control. Introducing the avatar as a 'character' on screen could have influenced children through mechanisms other than self-recognition—such as offering a mild sense of social presence or novelty-driven distraction. However, the similarity in cheating rates between the avatar condition (32.4% in Study 2) and online condition (35.1% in Study 1), and in gaze duration between the two conditions suggests that the avatar stimulus itself had limited influence on cheating behavior. This finding lends support to the interpretation that the observed differences were driven specifically by self-focused attention. The finding is consistent with Ding et al. (2019), which found that inducing other-awareness—by having young Chinese children view a peer’s photograph—did not significantly increase honest behavior relative to a control condition. The avatar in the present study similarly constitutes an other-oriented visual stimulus. Its failure to reduce cheating relative to the online condition aligns with the view that directing attention toward an external other, rather than the self, is insufficient to reduce dishonest behavior. Taken together, these findings suggest that directing attention to the participants effectively reduces cheating behavior.

## GENERAL DISCUSSION

The social environment worldwide changed dramatically due to the COVID-19 pandemic, and some academic activities continue to be conducted online. One negative consequence of online learning for university students was an increase in cheating during exams (Janke et al., 2021; Jenkins et al., 2023). We extended this line of research to examine online cheating among young children. Our findings showed that 5-year-olds cheated more frequently under online monitoring conditions. Importantly, we identified a method to reduce online cheating by promoting self-focused attention.

Our findings have several implications for future research. First, we revealed the specificity of children’s visual perspective-taking in online situations. Children in the current study generally understood the visual perspective of both online and offline proctors. This aligns with research showing that children of this age can understand others’ visual perspectives (Flavell et al., 1978, 1980; Frick et al., 2014). However, a notable exception was that children incorrectly believed the online proctor could see the center of the table, suggesting that camera-mediated perspective-taking presents unique challenges beyond traditional face-to-face contexts. The estimation of areas visible to proctors represents a crucial cognitive process in evaluating the risk of detection where over- or underestimating a proctor’s visual field may influence cost-benefit calculations and lead to different decisions. Further research is needed to explore the mechanisms underlying such perceptions and to examine their associated impact on variations in dishonest behavior.

Second, we confirmed that children cheat more in online situations where the proctor’s field of view is limited. In this condition, the likelihood of detecting dishonest behavior decreases, thereby lowering the costs associated with cheating. According to the cost-benefit framework, individuals are more likely to engage in behaviors when their relative costs decrease. Our perspective-taking task verified that children in each condition understood the monitoring constraints as intended—the online proctor’s limited and the offline proctor’s broader visual access. This suggests that children in the online condition perceived lower costs for engaging in cheating behavior relative to those in the offline condition. Under these conditions, children cheated significantly more in the online condition. While we cannot directly establish the mechanism linking children’s understanding of visual limitations to their cheating behavior, the present findings—that children demonstrated higher rates of cheating in the online condition where costs were lower—are consistent with the cost-benefit framework and suggest that children recognize and respond to differential monitoring constraints across contexts.

Third, we identified a new intervention to address online cheating among children. The effectiveness of the real-time self-image intervention can be explained by OSA theory (Duval & Wicklund, 1972). Notably, the reduction in cheating behavior observed when children viewed their real-time self-images mirrored the effect of human proctoring. Because real-time self-imaging is a default feature on many online platforms, it offers an easily implementable cost-effective and scalable alternative to human proctoring across educational contexts.

Future research should investigate online cheating and intervention strategies from a developmental perspective. We demonstrated that 5-year-old children cheat more in online conditions where monitoring is constrained, although it remains unclear whether this pattern is the same across other ages. To understand online cheating behavior, children must have VPT2, the ability to understand that another person may view the same object or scene differently. VPT2 typically emerges between the ages of four and five (Flavell et al., 1978, 1980; Frick et al., 2014). However, traditional face-to-face perspective-taking research has not addressed these issues in online contexts, and research on online visual perspective-taking remains limited. Therefore, key questions remain unresolved: When do children fully understand a proctor’s visual perspective in online situations? Children younger than five years may perceive a higher risk of detection, believing that the proctor can also see the answers, which might reduce their cheating. Conversely, older children more experienced with online learning and with a better understanding of online proctoring conditions may be more inclined to cheat. Further research is needed to clarify these developmental patterns.

Additionally, intervention strategies may be effective even at younger ages. Indeed, prior research has shown that mirror-based self-awareness inductions influenced honest behavior in children as young as 3 to 4 years old (Ding et al., 2019; Martin et al., 2020), suggesting that the prerequisites for such interventions may be met earlier in development than the age range examined in the current study. Regarding self-recognition, most 3-year-olds can already recognize themselves in real-time videos (Luxembourger et al., 2022; Suddendorf et al., 2007). Moreover, previous research indicates that even 3-year-olds have already internalized the standard (Schmidt et al., 2016; Schmidt & Tomasello, 2012). Children begin lying and cheating as early as 2 years of age (Evans & Lee, 2013). Thus, it may be necessary to examine whether the real-time self-image intervention is effective in children younger than those in the current study.

This study has some limitations. First, the online cheating observed among young children represents only one variant of adult online cheating. University students have been found to cheat through various methods, each with different contributing factors (Atoum et al., 2017; Noorbehbahani et al., 2022; Peytcheva-Forsyth et al., 2018). Nevertheless, because young children generally lack access to the technological tools that facilitate adult forms of cheating, their visual-perspective-taking abilities may represent a fundamental factor in online cheating behavior. Thus, our findings may capture the developmental roots of the monitoring-sensitive cheating observed in adults. Second, it remains unclear how real-time self-images affect cheating behavior. The limitations of our experimental setup prevented direct measurement of children’s attention to the avatar, although we did measure their attention to the screen. To examine the direct effects more precisely, future studies could use wearable eye-tracking technology.

Finally, the participants in the present study were mostly Japanese. Prior research has suggested that the impact of mirrors on cheating differs between Japan and Western societies. Heine et al. (2008) suggested that the presence of a mirror led North Americans to become more self-critical and less likely to cheat on a task, while there was no such impact for Japanese participants. Narita and Ishii (2020) replicated these findings and further revealed that hearing one’s own voice was more effective in reducing cheating than the presence of a mirror among Japanese participants. However, in the present study, the intervention using self-awareness proved effective among Japanese children.

The present study differed from previous research in two key aspects: participant age and the method of presenting participants’ images. While previous studies examined university students, the current study focused on 5-year-old children. This age difference may suggest that cultural differences in self-awareness may emerge later developmentally through accumulated experience. Furthermore, we used a real-time self-image display instead of a physical mirror. This methodological difference may have influenced self-awareness in ways that differ from mirror-based interventions. For instance, unlike mirrors, video displays can be positioned to remain within participants’ visual field during the task, potentially making the self-image more salient throughout the experimental procedure. Therefore, future research should examine OSA interventions from multiple perspectives, including cultural, developmental, and methodological aspects.

In conclusion, our findings highlight the developmental foundations of online academic cheating and offer insights for enhancing the quality of online education for young children.

## ACKNOWLEDGMENTS

We would like to thank M. Ishikawa for helping with data collection and M. Sakaguchi for assisting with data coding.

## FUNDING INFORMATION

This research was supported by a Grant-in-Aid for Scientific Research (A) to Y.K. (grant no. 24H00718). The funder played no role in the study design, data collection, analysis, publishing decisions, or manuscript preparation.

## AUTHOR CONTRIBUTIONS

M.O.: Conceptualization; Data curation; Formal analysis; Investigation; Methodology; Visualization; Writing – original draft. Y.K.: Conceptualization; Funding acquisition; Methodology; Project administration; Resources; Supervision; Writing – review & editing.

## DATA AVAILABILITY STATEMENT

All materials and anonymized data are available at https://osf.io/q8zta/.
